# Supplementary material for: No Association of Angiotensin-Converting Enzyme Insertion/Deletion (ACE I/D) Gene Polymorphism in the Susceptibility to Diabetic Retinopathy in Type 2 Diabetes Mellitus Patients: An Updated Meta-Analysis
Source: J Pers Med. 2023 Aug 26;13(9):1308. doi: 10.3390/jpm13091308 (PMC10533192; doi:10.3390/jpm13091308)
Supplement: Supplementary file 1 [file jpm-13-01308-s001.zip › jpm-2523914-supplementary.pdf]

To enable PROSPERO to focus on COVID-19 submissions, this registration record has undergone basic automated checks for eligibility and is published exactly as submitted. PROSPERO has never provided peer review, and usual checking by the PROSPERO team does not endorse content. Therefore, automatically published records should be treated as any other PROSPERO registration. Further detail is provided here.

Citation

Aline Pinho, Luciana Silveira, Angela Reis, Rodrigo Santos. Systematic reveiw of the correlation between the polymorphism rs1799752 of the Angiotensin Converting Enzyme gene and diabetic retinopathy. PROSPERO 2020 CRD42020215932 Available from:
https://www.crd.york.ac.uk/prospero/display_record.php?ID=CRD42020215932

Review question

What is the association betwen the rs1799752 Insertion/Deletion Angiotensin Converting Enzyme gene polymorphism and the occurrence of diabetic retinopathy in diabetic patients.

The Population are type 2 diabetic patients

The Exposure is the screening of the polymorphism rs1799752 of the Angiotensin Converting Enzyme gene

The Comparison are the polymorphisms I/I, I/D and D/D

The Outcome is the ocurrence or not of diabetic retinopathy

Searches

The search strategy will be designed to identify published and unpublished studies. An initial limited search of PubMed wiil be done to identify articles on this topic, followed by an analysis of the text words contained in the titles and abstracts, and the index terms used to describe those articles.

We will search the following electronic bibliographic databases: Embase, PubMed (MEDLINE) and The Cochrane Library

The grey literature will also be searched for published research and unpublished studies, using Google Scholar.

The search terms will combine "Polymorphism I/D", "ACE gene", "diabetic retinophathy", with the Boolean "AND".

Types of study to be included

Inclusion: Observational case-control studies published, no language restrictions, from 2015 to 2020 will be included in the review.

Exclusion: Other types of study, studies earlier than 2015.

Condition or domain being studied

Diabetic eye disease is a major complication of DM and causes visual impairment and blindness, with diabetic retinopathy being a leading cause of vision loss among working-age adults. The retinopathy is a progressive ophthalmic microvascular complication of diabetes characterised by the presence of microaneurysms, haemorrhages, exudates, venous changes, neovascularisation, and retinal thickening. If not treated it can lead to important vision loss and blindness.

Participants/population

People in any country in which a diagnosis with type 2 diabetes mellitus has been received according to the Word Health Organization (WHO).

Inclusion: Type 2 Diabetes for more than 10 years

Exclusion: People without diabetes or who were not evaluated for diabetic retinopathy

Intervention(s), exposure(s)

Screening of the polymorphism rs1799752 of the Angiotensin Converting Enzyme gene

Inclusion: People who were diagnosed with type 2 diabetes mellitus who were genotyped using the molecular analysis PCR technique to identify the rs1799752 Insertion/Deletion Angiotensin Converting Enzyme gene polymorphism and were evaluated for the ocorrence of diabetic retinopathy

Exclusion: People without diabetes or who were not evaluated for diabetic retinopathy

Comparator(s)/control

Comparison of the polymorphisms I/I, I/D and D/D

Context

Studies with patients with type 2 diabetic who were genotyped for the Angiotensin Converting Enzyme gene Insertion / Deletion polymorphism and screened for the presence of diabetic retinopathy.

Main outcome(s)

To determine genotype and allele frequencies;

To determine the probable association between Angiotensin Converting Enzyme gene I/D polymorphism and diabetic retinopathy;

To correlate the results of our study with the findings of different studies, thus facilitating discussion and conclusion.

Measures of effect

Odds Ratio

Additional outcome(s)

To determine characteristics about the groups, medium age and gender.

Measures of effect

Odds Ratio

Data extraction (selection and coding)

The strategy utilized three stages. Firstly a search was made in relation to the words contained in the topic at hand and the terms used to define the article. Secondly, all the keywords and terms identified in publications such as PubMed and Cochrane Library. The search of non-published material was acquired from Google Scholar. Third the reference lists of all reports and articles identified were saved for further study.

Risk of bias (quality) assessment

Studies selected for inclusion were assessed for methodological validity by two independent reviewers (L.C.S and V.O.) using standard the instruments for case-control studies from Joanna Briggs Institute Meta-Analysis of Statistics Assessment for Review Instrument (JBI-MAStaRI, University of Adelaide, Australia) http ://joannabriggs.org/assets/docs/critical-appraisal-tools/JBI_Critical_Appraisal-Checklist_for_Case_Control_Studies2017.pdf. The score determined for inclusion in the qualitative synthesis was ? 7

Strategy for data synthesis

All data was extracted by a single investigator using a standardized form. For each study, the citation details, sample size, frequency, details of diagnostic T2DM and DN. The primary outcome extracted was the individuals number each case group and control group. Secondarily the extracted results included were the respective quantitative for each genotype I/I, I/D, D/D both groups. Thus, the genotypic frequencies all studies includes were grouped into a single table and diversity assessed with use of the heterogeneity test (I^2) in contingency tables 2x2, to compare Odds ratio (OR) with confidence intervals (95%) determined in their respective studies. Thirdly, we evaluated the profile of individuals in each study regarding gender and age.

Analysis of subgroups or subsets

Analysis will be undetaken for both the case and control groups, for the presence or absence of retinopathy, and by genotype.

Contact details for further information

Victor Oriente
victororiente@gmail.com

Organisational affiliation of the review

Federal University of Goias (Medical School)
https://cienciassaude.medicina.ufg.br/

Review team members and their organisational affiliations [2 changes]

Mrs Aline Pinho. Federal University of Goias Medical School
Mr Luciana Silveira. Federal University of Goias Medical School
Mrs Angela Reis. Federal University of Goias Institute of Biological Science
Mr Rodrigo Santos. Federal University of Goias Institute of Biological Science

Type and method of review

Meta-analysis, Systematic review

Anticipated or actual start date

01 November 2020

Anticipated completion date [2 changes]

31 August 2022

Funding sources/sponsors

Federal University of Goias

Conflicts of interest

Language

English

Country

Brazil

Stage of review

Review Ongoing

Subject index terms status

Subject indexing assigned by CRD

Subject index terms

Diabetes Mellitus, Type 1; Diabetic Retinopathy; Humans; Peptidyl-Dipeptidase A; Polymorphism, Genetic

Date of registration in PROSPERO

12 December 2020

Date of first submission

11 November 2020

Stage of review at time of this submission [2 changes]

Stage	Started	Completed
Preliminary searches	Yes	Yes
Piloting of the study selection process	Yes	Yes
Formal screening of search results against eligibility criteria	No	No
Data extraction	No	No
Risk of bias (quality) assessment	No	No
Data analysis	No	No

Revision note

Change of the date by which the review is expected to be completed.

The record owner confirms that the information they have supplied for this submission is accurate and complete and they understand that deliberate provision of inaccurate information or omission of data may be construed as scientific misconduct.
The record owner confirms that they will update the status of the review when it is completed and will add publication details in due course.

Versions

12 December 2020
05 June 2022
16 June 2022
